# High Prevalence of MERS-CoV Infection in Camel Workers in Saudi Arabia

**DOI:** 10.1128/mBio.01985-18

**Published:** 2018-10-30

**Authors:** Abeer N. Alshukairi, Jian Zheng, Jingxian Zhao, Atef Nehdi, Salim A. Baharoon, Laila Layqah, Ahmad Bokhari, Sameera M. Al Johani, Nosaibah Samman, Mohamad Boudjelal, Patrick Ten Eyck, Maha A. Al-Mozaini, Jincun Zhao, Stanley Perlman, Abdulaziz N. Alagaili

**Affiliations:** a Department of Medicine, King Faisal Specialist Hospital and Research Center, Jeddah, Kingdom of Saudia Arabia; b Department of Microbiology and Immunology, University of Iowa, Iowa City, Iowa, USA; c State Key Laboratory of Respiratory Disease, Guangzhou Institute of Respiratory Health, The First Affiliated Hospital of Guangzhou Medical University, Guangzhou, Guangdong, China; d Department of Medical Research Core Facility and Platforms, King Abdullah International Medical Research Center, Riyadh, Kingdom of Saudi Arabia; e Department of Critical Care, King Saud Bin Abdulaziz for Health Sciences University, Riyadh, Kingdom of Saudi Arabia; f Department of Pathology and Laboratory Medicine, King Faisal Specialist Hospital and Research Center, Jeddah, Kingdom of Saudi Arabia; g College of Science and Health Professions, King Saud Bin Abdulaziz for Health Sciences University, Riyadh, Kingdom of Saudi Arabia; h Institute for Clinical and Translational Science, University of Iowa, Iowa City, Iowa, USA; i Department of Infection and Immunology, King Faisal Specialist Hospital and Research Center, Riyadh, Kingdom of Saudi Arabia; j Guangzhou Eighth People’s Hospital of Guangzhou Medical University, Guangzhou, China; k KSU Mammals Research Chair, Zoology Department, King Saud University, Riyadh, Kingdom of Saudi Arabia; Vanderbilt University Medical Center; Erasmus MC; Charité

**Keywords:** human Middle East respiratory syndrome, coronavirus, virus-specific antibody response, virus-specific T cell response, antibody, camel workers, Middle East respiratory syndrome, T cells

## Abstract

Middle East respiratory syndrome (MERS), a highly lethal respiratory disease caused by a novel coronavirus (MERS-CoV), is an emerging disease with high potential for epidemic spread. It has been listed by the WHO and the Coalition for Epidemic Preparedness Innovations (CEPI) as an important target for vaccine development. While initially the majority of MERS cases were hospital acquired, continued emergence of MERS is attributed to community acquisition, with camels likely being the direct or indirect source. However, the majority of patients do not describe camel exposure, making the route of transmission unclear. Here, using sensitive immunological assays and a cohort of camel workers (CWs) with well-documented camel exposure, we show that approximately 50% of camel workers (CWs) in the Kingdom of Saudi Arabia (KSA) and 0% of controls were previously infected. We obtained blood samples from 30 camel herders, truck drivers, and handlers with well-documented camel exposure and from healthy donors, and measured MERS-CoV-specific enzyme-linked immunosorbent assay (ELISA), immunofluorescence assay (IFA), and neutralizing antibody titers, as well as T cell responses. Totals of 16/30 CWs and 0/30 healthy control donors were seropositive by MERS-CoV-specific ELISA and/or neutralizing antibody titer, and an additional four CWs were seronegative but contained virus-specific T cells in their blood. Although virus transmission from CWs has not been formally demonstrated, a possible explanation for repeated MERS outbreaks is that CWs develop mild disease and then transmit the virus to uninfected individuals. Infection of some of these individuals, such as those with comorbidities, results in severe disease and in the episodic appearance of patients with MERS.

## Abstract (precis)

The Middle East respiratory syndrome (MERS) is a coronavirus (CoV)-mediated respiratory disease. Virus transmission occurs within health care settings, but cases also appear sporadically in the community. Camels are believed to be the source for community-acquired cases, but most patients do not have camel exposure. Here, we assessed whether camel workers (CWs) with high rates of exposure to camel nasal and oral secretions had evidence of MERS-CoV infection. The results indicate that a high percentage of CWs were positive for virus-specific immune responses but had no history of significant respiratory disease. Thus, a possible explanation for repeated MERS outbreaks is that CWs develop mild or subclinical disease. These CWs then transmit the virus to uninfected individuals, some of whom are highly susceptible, develop severe disease, and are detected as primary MERS cases in the community.

## INTRODUCTION

Middle East respiratory syndrome (MERS)-coronavirus (CoV), which emerged recently from zoonotic sources, causes severe pneumonia in patients in the Middle East and in travelers from this region (1–3). As of 30 June,2018, 2,229 cases with 791 deaths (35.5% case fatality rate) were reported to the WHO (http://www.who.int/emergencies/mers-cov/en/). A large fraction of MERS-CoV transmission occurs in health care settings, but approximately 30 to 45% of cases are considered primary (4, 5). It is generally accepted that dromedary camels, which are largely seropositive for MERS-CoV throughout the Arabian Peninsula, are the immediate source for most primary human infections (6–8). However, the majority of MERS patients report no camel contact, making the source of the infection uncertain (5, 9). One possible intermediary for human infection is camel workers (CWs), since this population has extensive and prolonged contact with these animals. MERS was not detected in humans until 2012, yet the virus has been circulating in camels at least since 1983, as assessed by serological tests (8, 10). Spread to humans may reflect recent intensification of camel herding in the Arabian Peninsula, resulting in increased camel-human interactions and enhanced human infection (11).

Confounding epidemiological studies, MERS-CoV-specific antibody responses are identified in many but not all infected patients and are only transiently detected in some patients, especially those with mild or asymptomatic infections (12, 13). Thus, epidemiological studies that rely on serological testing are predicted to underestimate the prevalence of the human infection. In contrast, virus-specific T cell responses may be longer lasting and thus, provide more accurate information about prevalence. In patients that recovered from the severe acute respiratory syndrome (SARS), virus-specific T cell responses could be detected as long as 11 years after infection, but antibody responses were not detected 6 years after infection (14, 15). Additionally, in a previous study (16), we reported the first analysis of MERS-CoV-specific T cell responses in MERS survivors and showed that T cell responses could be detected in some patients with undetectable antibody responses, especially those with mild or subclinical disease.

In this study, to address the possible role of CWs in MERS-CoV transmission, we analyzed virus-specific antibody and T cell responses in CWs with well-documented and extensive exposure to nasal and oral secretions while herding, capturing, and transporting camels in the Kingdom of Saudi Arabia (KSA). Unlike prior reports, we found that a high percentage of CWs had been previously infected with the virus, although most, if not all, had no clinical disease consistent with MERS. These results indicate that additional studies analyzing MERS-CoV transmission from CWs to non-CW contacts are warranted. The data also provide further support for the importance of measuring T cells, as well as antibody responses, to assess the prevalence of MERS and potentially that of other infectious diseases.

## RESULTS

### Clinical and demographic information.

**FIG 1:**
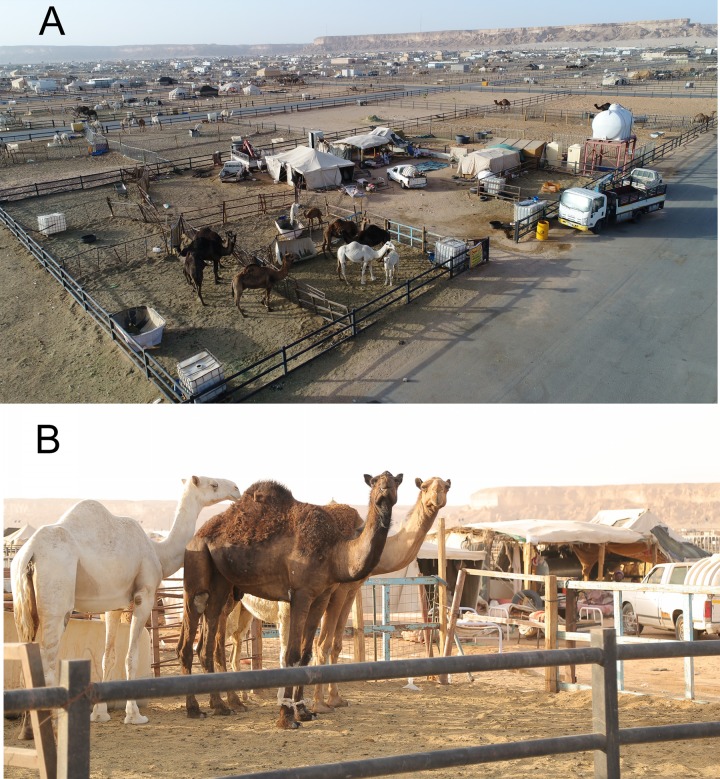
Riyadh New Camel Market in KSA. Photographs were taken from a drone (A) and at ground level (B). The photographs illustrate the close proximity between camels and the living quarters of CWs, possibly contributing to the high prevalence of MERS-CoV seropositivity in CWs. Image credit: A. N. Alagaili.

We obtained peripheral blood mononuclear cells (PBMCs) and sera from 30 CWs who worked in Riyadh New Camel Market (Fig. 1) and 44 healthy controls in KSA and Iowa, USA. Patient demographic and clinical information are shown in Table 1 and in more detail in Table S1. All of the study participants were adult males, and all CWs were exposed to camel oral and nasal secretions, urine, or both. CWs use their bare hands to control camels for transportation by turning the neck to one side of the body and inserting their hands inside the camel’s mouth. None used gloves or masks; some washed their hands after camel contact (Table 1 and Table S1). A minority had a history of fever or cold necessitating a visit to the emergency room within the previous 4 months. All subjects were expatriate workers (not Saudi Arabian citizens). Age, occupation, underlying medical conditions, history of tobacco use (smoking or chewing), and consumption of camel products (milk and meat) are also shown in the table.

**TABLE 1:** Characteristics of study participants

Category	Participant data^*b*^
Age (range [yrs])	37.7 (24–60)
Nationality, Saudi Arabian (no.)	0
Occupation^*a*^ (no.)	
Handler	21
Herder	9
Truck driver	12
Comorbidity (no.)	3
Handwashing after camel contact (no.)	22
Contact with MERS patient (no.)	0
Camel meat or milk consumption (no.)	15
Fever/cold within last 4 months (no.)	6
Tobacco use (no.)	15

a Some CWs had more than one role.

b *n* = 30.

10.1128/mBio.01985-18.2 TABLE S1 Characteristics of study participants (extended). Download Table S1, PDF file, 0.02 MB. Copyright © 2018 Alshukairi et al. 2018 Alshukairi et al. This content is distributed under the terms of the Creative Commons Attribution 4.0 International license.

### Serological testing of CWs.

We then measured MERS-CoV-specific antibody (Ab) titers in the sera of CW and healthy donors using enzyme-linked immunosorbent assay (ELISA), immunofluorescence assay (IFA), and 50% plaque reduction/neutralization titer (PRNT_50_) assay (Table 2). A total of 15/30 of CW sera had PRNT_50_ titers greater than 1:20 and were therefore considered positive. Of these 15 PRNT_50_ positive sera, 10 and 13 had positive or borderline ELISA and IFA titers, respectively. An additional CW serum had a positive ELISA and borderline IFA but a PRNT_50_ of <1:20 (CW13; Table 2). Notably, MERS-CoV-specific Ab levels were comparable to levels observed in survivors with mild or subclinical disease but lower than in those with severe disease (16). None of the healthy donors from KSA had serological evidence of infection as assessed by ELISA or PRNT_50_. Collectively, these results indicate that at least 50% of CWs had serological evidence of prior MERS-CoV infection.

**TABLE 2:** Serological test results

Case identifier	ELISA result	ELISA ratio	IFA result	IFA titer	PRNT_50_ titer
CW1^*a*^	Borderline	0.93	Positive	32	67.74
CW4^*a*^	Borderline	0.80	Positive	100	116.32
CW5^*a*^	Negative	0.14	Positive	32	58.56
CW7^*a*^	Borderline	0.89	Positive	32	91.26
CW10^*a*^	Negative	0.21	Borderline	10	23.46
CW15^*a*^	Borderline	0.82	Positive	32	51.69
CW16^*a*^	Borderline	0.90	Borderline	10	32.49
CW19^*a*^	Negative	0.53	Negative	<1:10	27.51
CW21^*a*^	Positive	1.60	Positive	32	60.21
CW23^*a*^	Negative	0.20	Positive	100	145.20
CW24^*a*^	Negative	0.21	Negative	<1:10	99.87
CW26^*a*^	Borderline	0.85	Borderline	10	30.10
CW27^*a*^	Borderline	0.86	Positive	32	51.69
CW28^*a*^	Positive	3.53	Borderline	10	31.82
CW30^*a*^	Borderline	0.83	Positive	32	83.40
CW13^*b*^	Positive	1.34	Borderline	10	<20
CW2^*c*^	Negative	0.20	Negative	<1:10	<20
CW3^*c*^	Negative	0.17	Negative	<1:10	<20
CW8^*c*^	Negative	0.23	Negative	<1:10	<20
CW9^*c*^	Negative	0.20	Negative	<1:10	<20
CW11^*c*^	Negative	0.30	Negative	<1:10	<20
CW12^*c*^	Negative	0.26	Negative	<1:10	<20
CW14^*c*^	Negative	0.28	Negative	<1:10	<20
CW17^*c*^	Negative	0.32	Negative	<1:10	<20
CW18^*c*^	Negative	0.53	Negative	<1:10	<20
CW20^*c*^	Negative	0.35	Negative	<1:10	<20
CW22^*c*^	Negative	0.25	Negative	<1:10	<20
CW25^*c*^	Negative	0.21	Negative	<1:10	<20
CW29^*c*^	Negative	0.17	Negative	<1:10	<20
CW31^*c*^	Negative	0.18	Negative	<1:10	<20
HD1-30^*d*^	Negative	0.13–0.18	ND^*e*^	ND	<20

a PRNT_50_ positive.

b ELISA-positive PRNT50 negative.

c ELISA negative, PRNT50 negative.

d KSA healthy donors.

e ND, not determined.

### Measurements of MERS-CoV-specific T cell responses.

**FIG 2:**
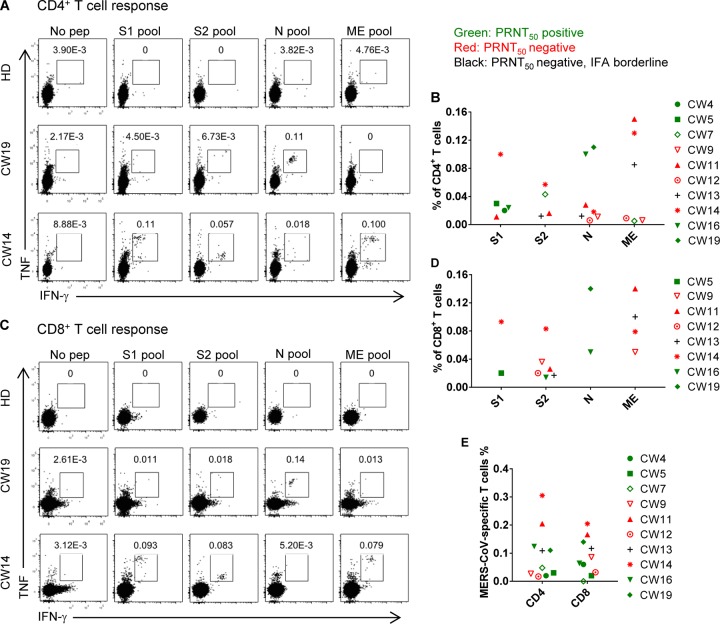
Virus-specific T cell responses are detected in some seronegative CWs. PBMCs from healthy donors and CWs were stimulated with MERS-CoV structural protein-specific peptide pools for 12 h in the presence of brefeldin A. Frequencies of MERS-CoV-specific CD4 (A and B) and CD8 (C and D) T cells (determined by IFN-γ and TNF intracellular staining) from seropositive (CW19) and seronegative (CW14) subjects are shown. (E) Summary of total T cell responses against all four peptide pools is shown.

Considering that antibody responses may be transient in MERS-CoV-infected patients with mild or asymptomatic disease (12, 13), we next measured virus-specific T cell responses to identify additional infected CWs. First, we determined the cellular composition of PBMCs (Table S2) using a previously described gating strategy (16; see also Fig. S1). Next, T cell responses were assessed using pools of overlapping 20-mer peptides covering all of the structural proteins (S1, S2, N, and ME, encompassing the N- and C-terminal portions of the spike [S] glycoprotein, the nucleocapsid [N] protein and the transmembrane [M] and envelope [E] proteins, respectively) (16). For these assays, peptides were used instead of virus-infected cells because MERS-CoV was shown to induce apoptosis in activated T cells, including virus-specific ones, after infection *in vitro* (17). We used these peptides in a series of intracellular cytokine (interferon-γ [IFN-γ] and tumor necrosis factor [TNF]) staining assays with PBMCs from CWs and healthy donors from the KSA and the USA (Fig. 2). Because T cell responses were relatively low, samples were counted as positive only if they dually expressed IFN-γ and TNF after peptide stimulation to maximize specificity.

10.1128/mBio.01985-18.1 FIG S1 Gating strategy for determining MERS-CoV-specific T cell responses. PBMCs from healthy donors and CWs were stimulated with MERS-CoV structural protein-specific peptide pools for 12 h in the presence of brefeldin A. MERS-CoV-specific CD4 and CD8 T cells were identified by IFN-γ and TNF double staining. Download FIG S1, PDF file, 0.1 MB. Copyright © 2018 Alshukairi et al. 2018 Alshukairi et al. This content is distributed under the terms of the Creative Commons Attribution 4.0 International license.

10.1128/mBio.01985-18.3 TABLE S2 PBMC cell composition. Download Table S2, PDF file, 0.02 MB. Copyright © 2018 Alshukairi et al. 2018 Alshukairi et al. This content is distributed under the terms of the Creative Commons Attribution 4.0 International license.

**FIG 3:**
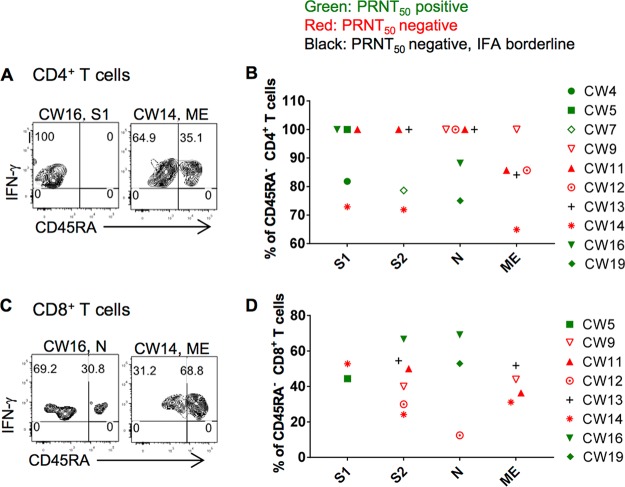
Memory phenotypes PBMC-derived MERS-CoV-specific T cells from CWs. PBMCs from seropositive (CW16) and seronegative (CW14) subjects were stimulated with MERS-CoV structural protein-specific peptide pools for 12 h in the presence of brefeldin A. IFN-γ^+^ TNF^+^ virus-specific CD4 (A, B) and CD8 (C, D) T cells were analyzed for CD45RA expression.

No CD4 or CD8 T cells from 22 healthy donors responded to the MERS-CoV peptide pools. PBMCs from 10 of 30 CWs contained CD4 or CD8 T cells that responded to at least one peptide pool. Of these 10 CWs, six were seropositive. We considered virus-specific T cell responses indeterminate in an additional 4 CWs, as described in Materials and Methods. Taken together, these data indicated that some seronegative CWs contained MERS-CoV-specific T cells and that the rate of MERS-CoV infection in CWs was greater than that revealed by serological testing alone. MERS-CoV-specific CD8 T cells from CWs were phenotypically a mixture of effector memory (CD45RA^−^) and effector (CD45RA^+^) cells (Fig. 3C and D), similar to the results obtained in analyses of MERS survivors (16). However, while MERS-CoV-specific CD4 T cells from some CWs were phenotypically effector memory (CD45RA^−^) cells, as described for all MERS survivors (16), cells from other CWs were CD45RA^+^, suggestive of the T_EMRA_ (effector memory T cells expressing CD45RA) subset.

### Risk factors for CW infection.

All of the CWs were exposed to camel oral and nasal secretions, and this exposure was the probable source for MERS-CoV infection. Next, we attempted to determine whether age, smoking, handwashing after camel contact, consuming camel meat or milk, specific occupation (camel truck driver, handler, or herder), or a recent history of fever or cold showed signs of a relationship with infection status. However, we were unable to identify any significant correlation between any of these factors and MERS-CoV infection (Table S3). Furthermore, groups of CWs live together. However, we found no evidence that infected individuals were preferentially domiciled together.

10.1128/mBio.01985-18.4 TABLE S3 Risk factors for MERS infection in Saudi Arabian CWs. Download Table S3, PDF file, 0.01 MB. Copyright © 2018 Alshukairi et al. 2018 Alshukairi et al. This content is distributed under the terms of the Creative Commons Attribution 4.0 International license.

## DISCUSSION

Although dromedary camels are considered to be the source for primary (nonnosocomial) human MERS, the majority of MERS cases do not describe camel exposure (9), making it critical to determine the precise pathway of camel to human transmission. Due to their close contact with camels and extensive exposure to camel mouth or nasal secretions and urine, CWs are at high risk for MERS-CoV infection and could serve as conduits for infection of the general population. Results from previous studies examining this issue were mixed (18–22), with some but not all describing increased MERS-CoV seropositivity in camel and abattoir workers. One study showed that camel shepherds and abattoir workers were preferentially infected with MERS-CoV compared to the general population, but only 2.4% and 3.6%, respectively, had serological evidence for infection (21). Here, using a cohort of CWs, we show that approximately 50% were seropositive for MERS-CoV. In addition, we documented virus-specific T cell responses in 4 seronegative CWs. In summary, a total of 20/30 CWs were identified as previously MERS-CoV infected when both antibody and T cell responses were measured. This rate of MERS-CoV infection is substantially higher than that reported previously, raising questions about whether we detected nonspecific responses, particularly cross-reactive ones to common human respiratory coronaviruses. Our results, which demonstrated no MERS-CoV-specific PRNT_50_ or T cell responses in healthy controls, are consistent with others that show only very low levels of MERS-CoV seropositivity in the general KSA population (21, 23). Furthermore, cross-neutralization with other human coronaviruses (HCoV) has not been reported previously, with the exception of one study that demonstrated low levels of anti-MERS-CoV neutralizing antibodies (≤1:20) in some severe acute respiratory syndrome (SARS) survivors (24). SARS-CoV no longer circulates in human populations, so prior infection with this virus is very unlikely to be the source for MERS immunopositivity. In addition, we considered neutralizing titers greater than 1:20 as positive, which should diminish issues of nonspecificity.

An unexpected result was the detection of MERS-CoV-specific CD45RA^+^ CD4 T cells (Fig. 3A and B). In our previous study, all virus-specific CD4 T cells were CD45RA^−^, consistent with an effector memory phenotype. CD45RA^+^ T cells were detected in the context of other human infections, such as those caused by dengue virus, human cytomegalovirus, and HIV, and were enriched for CD4 cytotoxic T lymphocytes (CTLs), which have been associated with better outcomes (25–29). The presence of CD45RA^+^ cells may contribute to milder disease in CWs.

Conclusions reached from previous studies of MERS-CoV infection of CWs were based on serological testing, with initial screening using a commercially available ELISA and confirmation by IFA and neutralization assay. In the present study, we also obtained a low seropositive rate based on ELISA (3 positive and 8 borderline). If only PBMC samples with positive ELISA had been further analyzed, the rate of positivity would have been no more than 10%, only a little higher than the values obtained in the prevalence study described above (21). On the other hand, IFA (9 positive and 5 borderline) and PRNT_50_ assays (15 positive) detected a greater number of infected CWs, while only one ELISA-positive serum was negative by PRNT_50_ assay. Together, these results suggest that the high rate of MERS-CoV infection reported here resulted from the selection of camel market-based CWs with well-documented exposure to camel nasal and oral secretions and to the measurement of PRNT_50_ and virus-specific T cell responses in all CWs. Camel markets, by facilitating herding and mixing of large numbers of animals in small enclosures, probably facilitated increased virus transmission both to MERS-CoV-naive camels and camel workers. This scenario is very similar to the one that occurred during the SARS epidemic, in which live-animal markets enhanced interspecies spread (30).

Our data also support the notion that using a multipronged approach to measuring antibody titers combined with assays for virus-specific T cell responses is useful for identifying subjects with mild or asymptomatic MERS and thereby better determining the incidence and prevalence of the infection. Inclusion of T cell assays identified an additional 25% of CWs as MERS-CoV infected compared to those identified by serological testing alone. We recognize that, at present, assessment of T cell responses is technically challenging, especially in countries in which blood is collected at elevated ambient temperatures, as occurs in camel habitats, and in which laboratory resources are limited. Thus, improvements in both virus-specific T cell assays and MERS-CoV-specific ELISAs will be required to obtain a more complete picture of the true prevalence of MERS.

In the first few years of the MERS epidemic, the vast majority of cases resulted from nosocomial transmission. Primary MERS cases now comprise a major fraction of new cases (4, 5), but how MERS-CoV is transmitted from camels to patients without evident camel contact is not well understood. Our results indicate that a high proportion of CWs are infected, and this, combined with data demonstrating that patients with subclinical MERS are contagious (31), suggests a plausible mechanism for how patients without documented camel exposure become infected. It is possible that some healthy contacts of CWs are subclinically infected, while exposed individuals with underlying comorbidities or who are otherwise more susceptible develop clinical disease.

Our results will need to be confirmed with larger numbers of CWs in prevalence studies and in longitudinal studies, in order to identify additional factors that contribute to transmission and to determine whether CWs are repeatedly infected. Also, camels are infected with other alphacoronaviruses and betacoronaviruses, including one that is related to HCoV-OC43 (32, 33). It will be important to determine whether these viruses infect humans, especially CWs, and if so, and while unlikely, whether these viruses induce a T cell response that is cross-reactive with that of MERS-CoV-specific T cells. Finally, it will be paramount to directly demonstrate infection of CW contacts to validate the notion that CWs are a source for spread within the community and to determine whether CWs are contagious after repeated exposure to the virus.

## MATERIALS AND METHODS

### Study design and participants.

The cohort for this study consisted of 30 CWs from Riyadh New Camel Market and 44 healthy controls recruited in Riyadh, KSA, and Iowa, USA. CWs were selected randomly, with the goal of including camel herders, handlers, and truck drivers. Camel herders feed, water, medicate, and milk camels. Camel handlers restrain, tighten (bind camels with ropes to prevent ambulation), and transport camels and have short but intense interactions with camels. Drivers help handlers and herders load camels into trucks. CWs control camels by placing their bare hands in the mouths of camels to avoid being bitten. Control samples of PBMCs were obtained from 14 anonymous donors at the University of Iowa and from 8 health care workers in KSA with no history of MERS exposure.

### Study approval.

The Institutional Review Board at King Faisal Specialist Hospital and Research Center, Jeddah (KFSHRC-J), KSA, approved the study. Written informed consent was obtained from all study participants.

### Clinical information.

CW demographic and clinical data were obtained by one of the corresponding authors (A. N. Alagaili) at the time of blood collection, using a written questionnaire.

### Virus and cells.

The EMC/2012 strain of MERS-CoV (passage 11, designated MERS-CoV) was provided by Bart Haagmans and Ron Fouchier (Erasmus Medical Center). All work with infectious MERS-CoV was conducted in the University of Iowa biosafety level 3 (BSL3) Laboratory.

### Serological testing.

Blood was collected from CWs in Riyadh New Camel Market (Fig. 1) at ambient temperatures as high as 45°C and was transported to King Abdullah International Medical Research Center in Riyadh. Temperatures were maintained at approximately 25°C during collection and transportation. PBMCs and sera were prepared from whole blood. Serum anti-MERS-CoV antibody titers were quantified by ELISA, immunofluorescence assay, and 50% plaque reduction/neutralization titer (PRNT_50_) assay (16). ELISA and IFA were performed as described previously, using commercially available kits (Euroimmun Medizinische Labordiagnotika AG) (16). The ELISA for MERS-CoV S-specific antibody was read as positive (>1.1), negative (<0.8), or borderline (0.8 to 1.1).

For 50% plaque reduction/neutralization titer (PRNT_50_) assays, serum samples were serially diluted in Dulbecco’s modified Eagle medium (DMEM) and mixed with an equal volume of MERS-CoV (EMC/2012) containing 80 PFU. Following incubation at 37°C for 1 h, aliquots were added to cultures of Vero 81 cells in 12-well plates and incubated at 37°C in 5% CO2 for 1 h. Virus titers were determined as previously described (16). Assays for PRNT_50_ were repeated at least two times for each serum sample, with identical results. We considered sera to be positive if the PRNT_50_ was greater than 1:20.

### Flow cytometry.

The following anti-human monoclonal antibodies were used: BD510-CD3 (HIT3a; BD Horizon), PerCP-Cy5.5-CD4 (RPA-T4; BioLegend), APC eFluor 780-CD8 (SK1; Invitrogen), APC-CD14 (M5E2; BioLegend), PE-CD19 (SJ25C1; BioLegend), PE-Cy7-CD56 (5.1H11; BioLegend), FITC-TCR γδ (B1; BD eBioscience), APC-IFN-γ (B27; BioLegend), PE-TNF (MAb11; Invitrogen), and PE-Cy7-CD45RA (HI100; BioLegend). Fc receptor-blocking solution was obtained from BioLegend.

PBMCs were prepared from blood using Lympholyte-H (Cedarlane) as per the manufacturer’s instruction. Cells were stored in liquid nitrogen prior to and during shipping to the University of Iowa, where the cells were analyzed. For surface staining, 10^5^ to 10^6^ cells were blocked with Fc receptor-blocking solution, labeled with live/dead staining dye (Thermo Fisher), and then stained with the indicated antibodies at 4°C. For *in vitro* intracellular cytokine staining, 4 × 10^5^ to 10 × 10^5^ cells/well were cultured in 96-well dishes at 37°C for 12 h in the presence of 2 μM peptide (GL Biochem, Shanghai) and brefeldin A (BFA; BD Biosciences). Cells were then labeled for cell surface markers, fixed/permeabilized with Cytofix/Cytoperm solution (BD Biosciences), and labeled with anti-cytokine antibodies. All flow cytometry data were acquired on a BD FACSVerse flow cytometer and analyzed using FlowJo software (Tree Star, Inc.). PBMCs were considered MERS-CoV experienced only if they expressed both IFN-γ and TNF in response to peptide stimulation. This stringent approach was used for two reasons. First, the fraction of cells expressing cytokines was lower than what we observed in our previous study of MERS survivors (16). In the case of some single-cytokine-expressing cells, levels were similar in the presence or absence of stimulation with peptide pools (e.g., Fig. 2A and C). Measuring only IFN-γ/TNF double expressers avoided this problem. Second, cell viability was less than 90%, likely reflecting exposure to high ambient temperatures (∼45°C) at the time of blood drawing. For these reasons, we were unable to definitively determine whether 4 CWs were positive or negative for a MERS-CoV-specific T cell response, so we classified them as indeterminate.

### Statistical analysis.

Fisher’s exact test was used to compare differences between groups, with *P* values of <0.05 considered statistically significant.
