# Supplementary material for: High Prevalence of MERS-CoV Infection in Camel Workers in Saudi Arabia
Source: mBio. 2018 Oct 30;9(5):e01985-18. doi: 10.1128/mBio.01985-18 (PMC6212820; doi:10.1128/mBio.01985-18)
Supplement: TABLE S1 [file mbo005184142st1.pdf]

Table S1-Characteristics of study participants-Extended

CW No.	Age	Occupation	Tobacco Use	Co-morbidities	Consumes camels products	Recent history of fever/cold	Handwashing after camel contact
1	48	Driver	Yes	No	Meat	No	Yes
2	40	Driver + handler	No	No	Milk	No	Yes
3	29	Driver	Yes	No	Meat	No	Yes
4	39	Herder	No	No	No	No	No
5	29	Herder	No	No	Milk	Yes	No
7	51	Driver + handler	No	Cardiovascular disease, diabetes, hypertension	Milk + meat	No	Yes
8	31	Herder	Yes	No	Milk	No	No
9	26	Handler	Yes	No	No	No	No
10	57	Herder	No	Hypertension	Milk	No	No
11	60	Herder + handler	No	Hypertension	No	Yes	No
12	42	Herder	No	No	Milk	No	No
13	37	Driver + handler	Yes	No	No	No	Yes
14	30	Handler	Yes	No	Milk + meat	No	Yes
15	44	Handler	No	No	No	No	Yes
16	41	Driver + handler	No	No	Milk + meat	Yes	Yes
17	32	Driver + handler	No	No	No	Yes	Yes
18	39	Handler	No	No	No	No	Yes
19	37	Handler	Yes	No	No	Yes	No
20	28	Driver + handler	Yes	No	Milk	No	Yes
21	28	Handler	No	No	No	No	Yes
22	60	Herder	No	No	No	No	Yes
23	25	Handler	No	No	No	No	Yes
24	44	Driver + handler	Yes	No	Milk + meat	Yes	Yes
25	43	Driver + handler	Yes	No	No	No	Yes
26	24	Driver + handler	Yes	No	No	No	Yes
27	52	Driver + handler	Yes	No	Milk	No	Yes
28	28	Handler	Yes	No	No	No	Yes
29	23	Herder	No	No	No	No	Yes
30	35	Handler + herder	Yes	No	Milk	No	Yes
31	29	Handler	Yes	No	Milk	No	Yes
