# Supplementary material for: High Prevalence of MERS-CoV Infection in Camel Workers in Saudi Arabia
Source: mBio. 2018 Oct 30;9(5):e01985-18. doi: 10.1128/mBio.01985-18 (PMC6212820; doi:10.1128/mBio.01985-18)
Supplement: FIG S1 [file mbo005184142sf1.pdf]

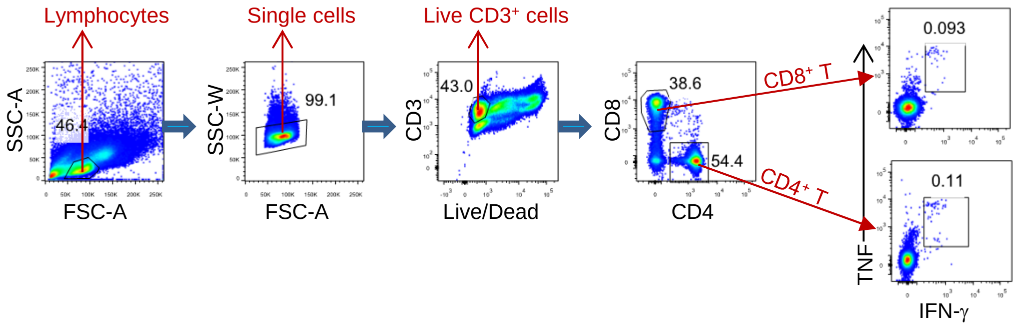

Supplementary Figure 1. Gating strategy for determining MERS-CoV-specific T cell responses. PBMCs from healthy donors and CWs were stimulated with MERS-CoV structural protein-specific peptide pools for 12 hours in the presence of brefeldin A. MERS-CoV-specific CD4 and CD8 T cells were identified by IFN- γand TNF double staining.
