# Supplementary material for: High Prevalence of MERS-CoV Infection in Camel Workers in Saudi Arabia
Source: mBio. 2018 Oct 30;9(5):e01985-18. doi: 10.1128/mBio.01985-18 (PMC6212820; doi:10.1128/mBio.01985-18)
Supplement: TABLE S2 [file mbo005184142st2.pdf]

Table S2-PBMC cell composition

Case ID	% CD3/LC	% CD4/LC	% CD8/LC	% GDT/LC	% NKT/LC	% CD19/LC	% NK/LC	% CD14/PBMC
Camel workers								
CW1	73.1	36.2	28.9	0.62	4.04	14.2	8.96	2.43
CW2	76	39.8	32.5	2.88	1.16	12.4	7.65	2.41
CW3	69.7	34.2	27.4	0.16	5.33	14.7	11.4	4.82
CW4	60.4	21.8	32.3	1.79	5.75	18.1	16.3	7.16
CW5	77.7	19.6	49.9	6.93	3.49	10.8	6.07	4.5
CW7	53.7	32.4	14.6	0.61	1.62	28.9	11.3	1.68
CW8	65.5	28.4	31	2.53	1.16	13.7	15.2	2.42
CW9	58.1	19.2	32.8	1.08	3.21	14.5	22.6	2.8
CW10	67	20.9	39.8	0.86	10.2	9.22	16.5	2.1
CW11	58.2	21.5	31.6	0.31	1.56	27	9.95	3.65
CW12	71.5	17.8	39.1	3.57	14	15.7	8.22	3.13
CW13	71.8	18.9	45.9	3.15	7.68	14.9	9.3	4.24
CW14	59.5	19.4	32.8	0.69	2.89	15.6	18.1	6.29
CW15	78.2	21.2	41.4	3.79	5.84	7.12	10.5	0.37
CW16	58.2	24.2	25.3	2.85	6.86	14.6	20	4.11
CW17	73.3	15	52.6	0.88	3.8	13.7	7.69	2.44
CW18	74.3	21.6	47.3	1.8	6.51	11.1	7.88	3.34
CW19	59.4	25.9	27.4	1.28	3.19	16.5	19.2	1.61
CW20	62.4	17.9	40.8	0.1	2.13	17	13.9	6.16
CW21	68.3	17	43.8	5.55	7.43	10.4	16.4	2.73
CW22	50.3	32.1	13.6	0.9	1.17	18	23.7	5.94
CW23	78.9	36.6	33.9	6.67	2.01	8.97	6.89	0.47
CW24	65.4	16.3	41.1	8.35	4.22	20.2	8.84	3.47
CW25	74.2	22.5	41	4.92	12.7	8.81	14.3	2.22
CW26	60.9	18.9	36.3	3.51	2.21	16	15.6	4.94
CW27	63.8	25.5	33.3	5.25	6.03	20.5	11.5	2.74
CW28	68.6	28	37.3	3.53	2.06	16.3	9.42	2.07
CW29	68.2	25.5	37	5.22	1.52	13.9	12.7	2.64
CW30	72.4	30.4	38.6	2.89	4.65	12.9	10.5	2.82
CW31	72.2	18.4	50.8	2.27	0.32	10.9	8.52	1.93
Healthy donors*								
HD1	87.6	46.2	18.4	0.068	5.15	5.7	7.1	5.22
HD2	74.5	36.5	17.1	0.024	0.89	13	10.6	28.4
HD3	61.1	36.8	22.2	1.54	1.8	15.1	15	8.12
HD4	73.5	37.9	21.8	1.15	1.11	13	12.7	8.77
HD5	75.4	40.3	24.5	1.77	3.36	10.4	8.96	7.99
HD6	65.6	51.7	12.4	1.13	0.36	18.2	4.77	5.3
HD7	67.2	52.9	12.9	0.79	0.61	15.3	5.28	4.27
HD8	56	35.8	15.7	0.92	3.57	20.4	9.36	8.16
HD9	62.9	40.5	20.3	1.05	10.8	22.37	4.35	3.35
HD10	50.7	34.5	9.02	1.3	4.92	20.15	10.11	10.25
HD11	59.6	36.5	11.7	1.22	7.88	23.4	2.53	7.69
HD12	67.5	41.7	20.17	2.13	3.26	25.38	7.98	8.52
HD13	68.4	48.7	11.3	0.55	4.35	21.45	9.24	11.3
HD14	70.6	50.1	17.1	0.97	2.64	16.52	8.16	14.2
HD15	62.6	42.6	11.8	1.15	3.67	20.31	14.59	7.35
HD16	67.2	43.2	10.54	0.76	4.32	18.6	10.17	13.6
HD17	76	40.2	33	0.58	2.21	7.23	8.52	15.8
HD18	68.5	39.2	25.4	1.17	2.55	17.93	11.2	9.32
HD19	64.8	46.2	11.8	2.08	5.43	23.7	10.52	10.16
HD20	79.4	61	18.06	0.23	0.15	9.83	7.62	15.6
HD21	60.3	43.2	12.46	1.32	4.25	22.57	15.34	7.32
HD22	72.8	48.1	17.6	0.89	3.27	17.65	9.28	5.28

*HD1-14-USA (blue); HD15-22-KSA (green)
