# Supplementary material for: High Prevalence of MERS-CoV Infection in Camel Workers in Saudi Arabia
Source: mBio. 2018 Oct 30;9(5):e01985-18. doi: 10.1128/mBio.01985-18 (PMC6212820; doi:10.1128/mBio.01985-18)
Supplement: TABLE S3 [file mbo005184142st3.pdf]

Table S3: Risk factors for MERS infection in Saudi Arabian CWs			
Characteristic	[1]MERS-CoV+ (20)	MERS-CoV- (10)	*p*-value
Occupation			
Handler	13 (0.65)	7 (0.7)	1.0
Herder	8 (0.4)	2 (0.2)	0.42
Truck driver	7 (0.35)	6 (0.6)	0.25
Co-morbidity	3 (0.15)	0 (0.0)	0.53
Handwashing after camel contact	13 (0.65)	9 (0.9)	0.21
Camel meat or milk consumption	10 (0.5)	5 (0.5)	1.0
Fever/cold within last 4 months	5 (0.25)	1 (0.10)	0.63
Tobacco use	5 (0.25)	5 (0.5)	0.23

[1]Positive for MERS-CoV immune response (antibody, T cell or both).
